# Intraoperative blood salvage may shorten the lifespan of red blood cells within 3 days postoperatively: A pilot study

**DOI:** 10.1097/MD.0000000000008143

**Published:** 2017-09-29

**Authors:** Xin-Yi Liao, Shan-Shan Zuo, Wen-Tong Meng, Jie Zhang, Qin Huang, Da-Ming Gou

**Affiliations:** a Department of Anesthesiology, Affiliated Hospital of Zunyi Medical University, Zunyi; b Department of Anesthesiology, Zhengzhou Central Hospital, Zhengzhou, Henan; c Laboratory of Stem Cell Biology, State Key Laboratory of Biotherapy; d Key Laboratory of Transplant Engineering and Immunology, Ministry of Health, West China Hospital, Sichuan University, Chengdu; e Department of Anesthesiology, Hospital of Honghuagang District, Zunyi, Guizhou, China.

**Keywords:** intraoperative blood salvage, RBC lifespan, RBC-engulfing granulocyte

## Abstract

**Background:**

Intraoperative blood salvage (IBS) recovers most lost blood, and is widely used in the clinic. It is unclear why IBS does not reduce long-term postoperative requirements for red blood cells (RBCs), and 1 possibility is that IBS affects RBC lifespan.

**Methods:**

Prospectively enrolled patients who underwent spine, pelvic, or femur surgery not involving allogeneic RBC transfusion were grouped based on whether they received IBS or not. Volumes of blood lost and of RBCs salvaged during surgery were recorded. Total blood cell counts, levels of plasma-free hemoglobin, and CD235a-positive granulocytes were determined perioperatively.

**Results:**

Although intraoperative blood loss was higher in the IBS group (n = 45) than in the non-IBS group (n = 52) (*P* < .001), hemoglobin levels were similar between groups (*P* = .125) at the end of surgery. Hemoglobin levels increased in non-IBS patients (4 ± 11 g/L), but decreased in IBS patients (−7 ± 12 g/L) over the first 3 postoperative days. Nadir hemoglobin levels after surgery were higher in the non-IBS group (107 ± 12 g/L) than in the IBS group (91 ± 12 g/L). Salvaged RBC volume correlated with hemoglobin decrease (*r* = 0.422, *P* = .004). In multivariate analysis, salvaged RBC volume was an independent risk factor for hemoglobin decrease (adjusted odds ratio 1.002, 95% confidence interval 1.001–1.004, *P* = .008). Flow cytometry showed the numbers of CD235a-positive granulocytes after surgery to be higher in the IBS group than in the non-IBS group (*P* < .05).

**Conclusion:**

IBS may shorten the lifespan of RBCs by triggering their engulfment upon re-infusion (China Clinical Trial Registry ChiCTR-OCH-14005140).

## 1 Introduction

Intraoperative blood salvage (IBS), which allows recovery of most blood lost during surgery, is widely used in the clinic. However, whether it reduces postoperative requirements for allogeneic red blood cells (RBCs) is unclear. A multicenter cohort study^[1]^ showed IBS to be associated with lower blood transfusion during revision hip surgery; similar results were reported for patients undergoing cardiac surgery^[2]^ or elective infrarenal aortic surgery.^[3]^ In contrast, another study^[4]^ found that IBS significantly reduced transfusions of packed RBCs within 24 hours after cardiac surgery, but not during the subsequent hospital stay. In addition, large, multicenter randomized controlled trials showed that IBS during primary hip or knee replacement surgery did not decrease the mean number of allogeneic RBCs transfused or the proportion of patients receiving transfusions after surgery.^[5–7]^

The available evidence, then, suggests that IBS does not provide substantial benefits over longer postoperative periods. The reasons for this are unclear. Since IBS has been shown to reduce the deformability of RBCs, and also their content of 2,3-diphosphoglycerate,^[8]^ it is possible that IBS damages RBCs. Indeed, our group has shown that IBS significantly reduces adenine nucleotide levels, causing RBCs to become stomatocytes and even “ghosts” at 48 hours after surgery.^[9]^ Damaged RBCs should be rapidly eliminated after being returned to the patient, which may explain why IBS does not reduce postoperative blood requirements in the longer term.

One possibility is that damaged RBCs undergo rupture (hemolysis) or are engulfed by phagocytes via receptor-mediated interactions, leading the RBCs to be cleared from the body. During engulfment, phagocytes may recognize phosphatidylserine exposed on the surface of the RBCs.^[10,11]^ The present study tested this hypothesis by examining whether and how IBS shortens RBC lifespan during the first 3 days after surgery.

## 2 Methods

### 2.1 Patients

Patients scheduled for spine, pelvic, or femur surgery at the Affiliated Hospital of Zunyi Medical University were prospectively enrolled between January 1, 2015 and November 30, 2015 if they were between 18 and 70 years old and had American Society of Anesthesiologists physical status I to III. Patients were excluded if they had one of the following conditions: a serious disorder of the coronary, peripheral, and/or carotid arteries; low hemoglobin levels, defined as <8 g/dL; hematologic or malignant disease; diabetes; untreated hypertension, defined as diastolic blood pressure >95 mm Hg; renal or liver dysfunction; sickle cell anemia; or prophylactic anticoagulant therapy. They were also excluded if they received allogeneic RBC transfusion, or if they were undergoing repeat or emergency surgery. Patients exited the study if they withdrew consent after enrollment.

This study was approved by the Ethics Committee of Zunyi Medical University, and has been registered in the China Clinical Trial Registry (ChiCTR-OCH-14005140).

### 2.2 Anesthesia, IBS, and study design

All patients received general anesthesia involving intravenous induction with midazolam, sufentanil, propofol, and muscle relaxants, followed by endotracheal intubation and positive-pressure ventilation. Anesthesia was maintained by sevoflurane inhalation, sufentanil, propofol, and muscle relaxants.

For all patients enrolled in our study, blood was salvaged during surgery using an autologous blood recovery system (3000P, Jingjing Medical Equipment, Beijing, China), as described.^[9]^ Briefly, a negative pressure system (−4.0 to −6.0 kPa) collected blood lost from the time of skin incision until wound closure. Heparin (25 U/mL) was added to 0.9% normal saline to prevent coagulation. RBCs were washed in 250-mL centrifuge bottles until the supernatant was clear, which required 1.5 to 2 L of solution. If >250 mL of RBCs were salvaged, they were re-infused into the patient as soon as possible. If <250 mL were salvaged, the RBCs were not re-infused into the patient. Patients who received transfusion of salvaged RBCs were assigned to the IBS group, and patients who did not receive salvaged RBCs were assigned to the non-IBS group. We estimated that the smallest acceptable sample size would be 36 patients in each group, to have 80% likelihood of detecting a difference of 1 g/dL in hemoglobin levels between the 2 groups on the first day after surgery. This power calculation assumed a standard deviation of 15 g/L in hemoglobin level measurements, and a 2-sided alpha level of 5%.

### 2.3 Blood loss measurement and transfusion

Blood loss in the operating room was assessed by measuring blood volume in the suction bottle (volume = total volume − volume of normal saline containing anticoagulant), and also by weighing blood-stained absorbent gauzes generated between skin incision and skin closure (assuming blood density of 1 g/mL). Blood loss after surgery was defined as the volume of blood shed into the drainage tube over the first 72 hours after surgery.

Decisions to give blood product transfusions were made in the operating room by the anesthesiologist and in the ward by the surgeon. The threshold hemoglobin concentration for performing transfusion of packed RBCs was 7 g/dL.

### 2.4 Standard hematology indices

Blood cell counts and hemoglobin levels were determined using an automatic blood analyzer before surgery, at the end of surgery, and at 24, 48, and 72 hours after surgery. These time points were chosen because our preliminary studies (data not shown) indicated that RBC counts were stable by 3 days after surgery. At the same time points, levels of free hemoglobin in plasma were determined using enzyme-linked immunosorbent assay (Cayman Chemical Company) according to the manufacturer's instructions. Counts of CD235a-positive granulocytes and monocytes were determined using flow cytometry. Requirements for blood production during surgery and during the first 3 postoperative days were recorded.

### 2.5 Flow cytometry assay for CD235a-positive phagocytes

In this procedure, blood (1.0 mL) was lyzed with ammonium chloride (9 mL) and the cells were washed twice with phosphate-buffered saline (PBS), suspended in PBS, and counted using a hemacytometer. Cell suspensions (50.0 μL, 1 × 10^6^ cells) were incubated with 3.0 μL PE-conjugated anti-human CD14 antibody (TK4, Genetex) for 30 minutes at 4°C in the dark. The mixture was centrifuged at 600*g* for 7 minutes at 4°C, and cells were washed twice with PBS, then resuspended in 250.0 μL fixation/permeabilization solution (Cytofix/Cytoperm Plus, BD Bioscience, San Jose, CA) at 4°C for 20 minutes. Cells were washed twice in 1 × BD Perm/Wash buffer. Fixed/permeabilized cells were thoroughly resuspended in 50 μL BD Perm/Wash buffer containing 3.0 μL fluorescein isothiocyanate (FITC)-conjugated mouse anti-human CD235a antibody (BD Bioscience) and incubated at 4°C for 30 minutes in the dark. IgG1-FITC/PE served as a negative control. Cell pellets were washed twice with 1× BD Perm/Wash buffer, suspended in PBS, then analyzed by flow cytometry.

**Figure 1:**
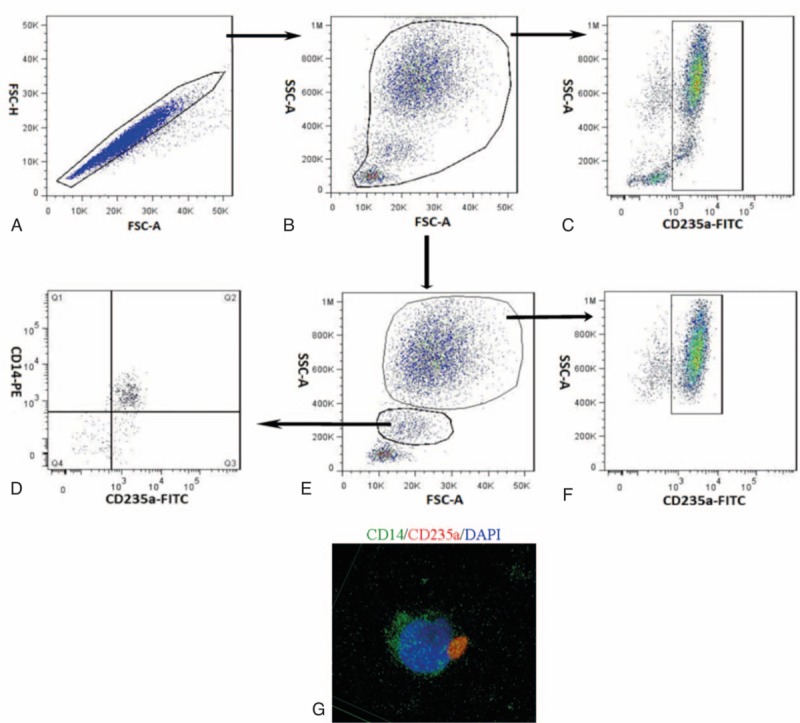
Flow cytometry to determine CD235a-positive granulocytes and monocytes in blood from patients. (A) Aggregated cells in blood samples were excluded based on dot plots of forward scatter (FSC) height versus FSC area. (B) Platelets and cell debris were excluded based on dots plots of FSC versus side scatter (SSC). (C) CD235a-positive white blood cells were gated. (D) Granulocytes and monocytes were gated separately. (E) CD235a-positive monocytes are shown. (F) CD235a-positive granulocytes are shown. (G) Representative confocal fluorescence micrographs showing CD14-positive cells (green) and CD235a-positive cells (red).

At least 50,000 events were acquired on a FACSAria cytometer (BD Biosciences), and data were analyzed using Flowjo software (Tree Star). A sequential gating strategy was used during analysis, and CD235a-positive phagocytes were confirmed using confocal microscopy (Fig. 1).

### 2.6 Binding of Annexin-V by RBCs

Preoperative arterial blood (fresh RBCs) and salvaged RBCs from 10 patients in the IBS group were centrifuged at 800*g* for 5 minutes. The supernatant was discarded and the cell pellet was resuspended in PBS to a concentration of 5 × 10^6^ cells/mL. Cells (5 × 10^6^) were seeded into three 6-cm centrifuge tubes. The cells were plated and cultured at 37°C on a platform rotating at 80 rpm. To determine what fraction of these cells bound Annexin-V, cells were harvested after 0, 24, 48, or 72 hours of culture, and diluted to 1 × 10^6^ cells/mL using Hank balanced salt solution (Invitrogen). To the resuspended cells (100.0 μL, 1 × 10^5^ cells) was added 3.0 μL allophycocyanin-conjugated Annexin V (BD Bioscience), and the mixture was incubated at room temperature for 15 minutes in the dark. To this mixture was added 400.0 μL 1× binding buffer, and then flow cytometry of at least 50,000 events was performed.

### 2.7 Statistical analysis

Data were analyzed using SAS 9.1 (SAS Institute, Cary, NC). Continuous measurements were reported as mean ± standard deviation, and differences between groups were assessed for significance using Student *t* test. Categorical measurements were expressed as counts (percentages), and Fisher exact tests were used to assess differences between groups. The paired Student *t* test was used to assess the significance of differences in the proportion of fresh and salvaged RBCs that bound Annexin-V after incubation. Decreases in hemoglobin levels were calculated by subtracting the nadir value (measured within 3 days after surgery) from the values at the end of surgery. Pearson correlation analysis was used to detect relationships of salvaged volume with decreases in levels of hemoglobin within 3 days after surgery. Univariate and multivariate logistic analyses were used to analyze the association of baseline or intraoperative variables with either intraoperative blood loss or decrease in hemoglobin levels. Odds ratios (ORs) and associated 95% confidence intervals (95% CIs) were calculated to predict the change in outcome variable per unit increase of the predictor. The threshold for statistical significance was defined as *P* < .05.

## 3 Results

**Figure 2:**
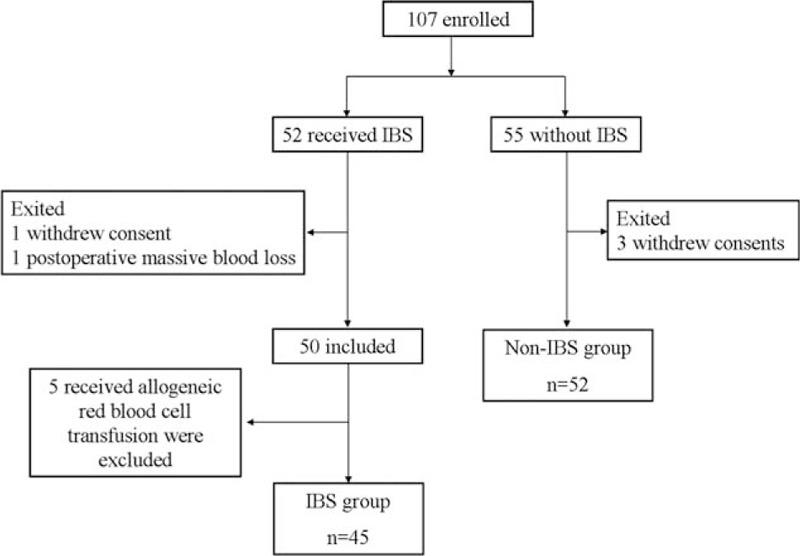
Patient flowchart. IBS = intraoperative blood salvage.

In all, 107 patients were assessed for enrollment in the study, of whom 52 received transfusion with salvaged RBCs. Of these 52 patients, 2 exited the study because 1 withdrew consent and another had a postoperative accident that led to massive blood loss. Another 5 were excluded because they received transfusions of altogether 21 units of allogeneic packed RBCs, with 10 units (47.6%) transfused in the operating room, 3 units (14.3%) transfused on postoperative day 1, 4 units (19.0%) on day 2, and 4 units (19.0%) on day 3. These patients were excluded to avoid confounding effects of allogeneic RBCs on analysis of salvaged RBC survival. The remaining 45 patients were included in the final analysis (Fig. 2, Table 1).

**Table 1:**
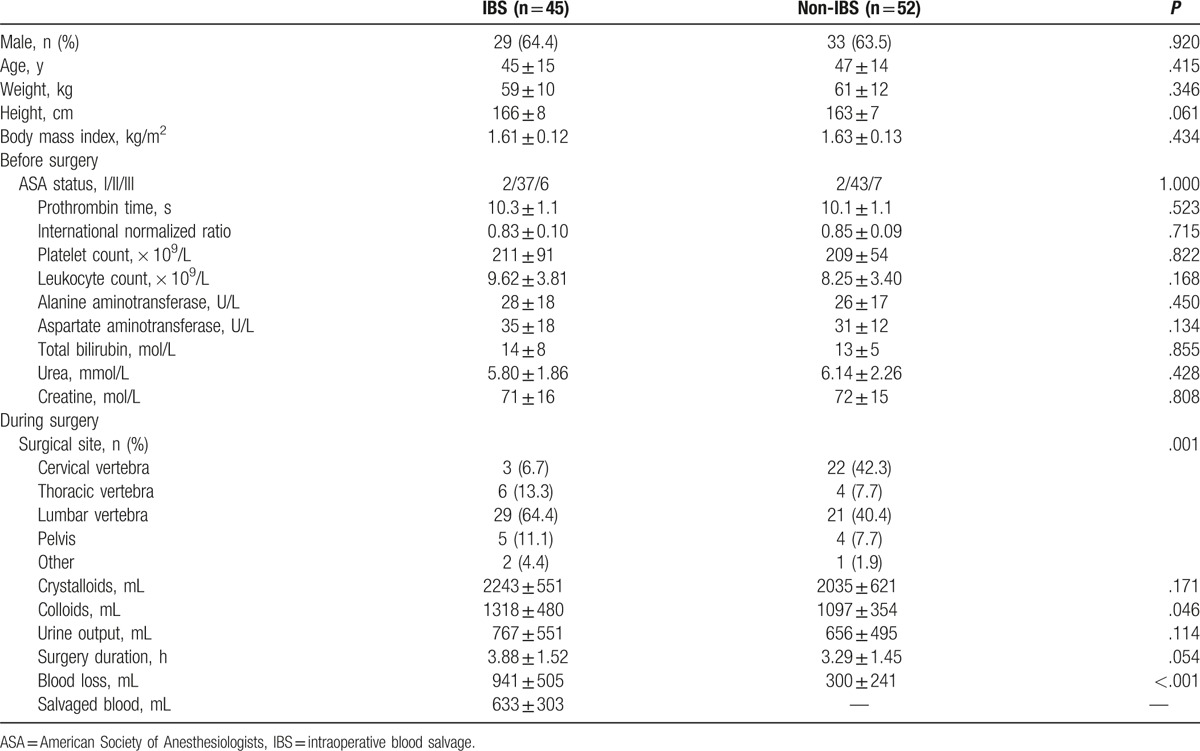
Perioperative characteristics of patients.

Of the 55 patients who did not receive transfusion of salvaged RBCs, 3 exited the study because they withdrew consent. In the end, 52 patients in the non-IBS group were included in the final analysis.

### 3.1 Blood loss, blood salvage, and transfusion

**Figure 3:**
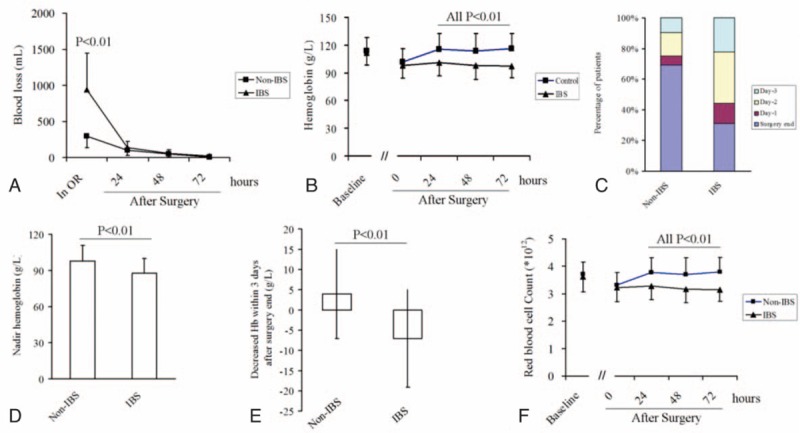
Blood loss, perioperative hemoglobin levels, and their changes. (A) Volume of blood lost during and after surgery. (B) Hemoglobin levels at the end of surgery were similar between groups, but the levels over the first 3 postoperative days were higher in the non-IBS group than in the IBS group. (C) The nadir hemoglobin level was reached by most patients at the end of surgery in the non-IBS group, but between postoperative days 1 to 3 in the IBS group. (D) Nadir hemoglobin levels were higher in the non-IBS group than in the IBS group. (E) Decreases in hemoglobin levels were calculated by subtracting nadir levels within 3 days of surgery from the levels at the end of surgery. (F) Results for red blood cell counts were similar to those for hemoglobin levels.

The proportion of patients undergoing surgery at sites of lumbar vertebra was higher in the IBS group than in the non-IBS group. Surgery duration was slightly longer and operative colloid requirement was greater in the IBS group (Table 1). Among all patients, blood loss was 552 ± 472 mL (median 450 mL; interquartile range 245–740 mL), and it correlated with surgical duration (*r* = 0.45, *P* < .001). In univariate logistic analysis, surgical duration was associated with massive blood loss, defined as loss of at least 500 mL (OR 1.94, 95% CI 1.34–2.82, *P* < .001). This relationship remained significant after adjusting for age, sex, body mass index, prothrombin time, international normalized ratio, platelet count, and surgical site (adjusted OR 1.96, 95% CI 1.22–3.13, *P* = .005). Intraoperative blood loss was higher in the IBS group than in the non-IBS group (Table 1, Fig. 3A).

The total volume of salvaged RBCs was 633 ± 303 mL for IBS patients, corresponding to salvage of approximately 67% of lost blood. A strong correlation was found between the volume of blood lost and the volume of salvaged RBCs (*r* = 0.84, *P* < .001).

### 3.2 Hemoglobin levels and IBS

Hemoglobin levels were similar between the 2 groups before surgery (*P* = .575) and at the end of surgery (*P* = .317), with hemoglobin decreasing in both groups between these 2 time points (*P* < .001). The magnitude of this decrease was similar between the non-IBS group (from 116 ± 15 to 102 ± 14 g/L, 12% decrease) and the IBS group (from 114 ± 12 to 98 ± 14 g/L, 14% decrease). Hemoglobin levels in the non-IBS group returned to presurgery levels by day 1 after surgery (116 ± 17 g/L; *P* = .877), and they remained stable until at least 72 hours after surgery. In contrast, hemoglobin levels on day 1 after surgery in the IBS group (101 ± 14 g/L) were still significantly below the presurgery baseline (*P* < .001), and they decreased further on days 2 and 3 after surgery. On all 3 days after surgery, hemoglobin levels were significantly lower in the IBS group than in the non-IBS group (Fig. 3B).

Most non-IBS patients (69%, 36 of 52) reached their nadir hemoglobin levels at the end of surgery, whereas most IBS patients (69%) reached their nadir hemoglobin levels between day 1 and day 3 after surgery (Fig. 3C). Nadir hemoglobin levels after surgery were higher in the non-IBS group (107 ± 12 g/L) than in the IBS group (91 ± 12 g/L; *P* = .001) (Fig. 3D).

To determine the impact of intraoperative blood salvage on hemoglobin levels after surgery, hemoglobin decreases were calculated by subtracting the nadir levels during the 3 days after surgery from the levels at the end of surgery. Hemoglobin levels increased in non-IBS patients (4 ± 11 g/L), but decreased in IBS patients (−7 ± 12 g/L) within 3 postoperative days (Fig. 3E). Pearson correlation analysis found a significant relationship between volume of salvaged RBCs and hemoglobin decrease (*r* = 0.422, *P* = .004). Univariate logistic regression found hemoglobin decrease to be associated with salvaged RBC volume (OR 1.002, 95% CI 1.001–1.003, *P* = .004) and surgery site (*P* = .005). Volume of salvaged RBCs was an independent risk factor for hemoglobin decrease (adjusted OR 1.002, 95% CI 1.001–1.004, *P* = .008), even after adjusting for age, sex, body mass index, prothrombin time, international normalized ratio, platelet, surgical time, and surgery site. Similar results were observed for RBC counts (Fig. 3F).

Mean corpuscular volume and mean corpuscular hemoglobin were similar between the groups during the observation period.

### 3.3 CD235a-positive granulocytes and IBS-induced phosphatidylserine exposure

**Figure 4:**
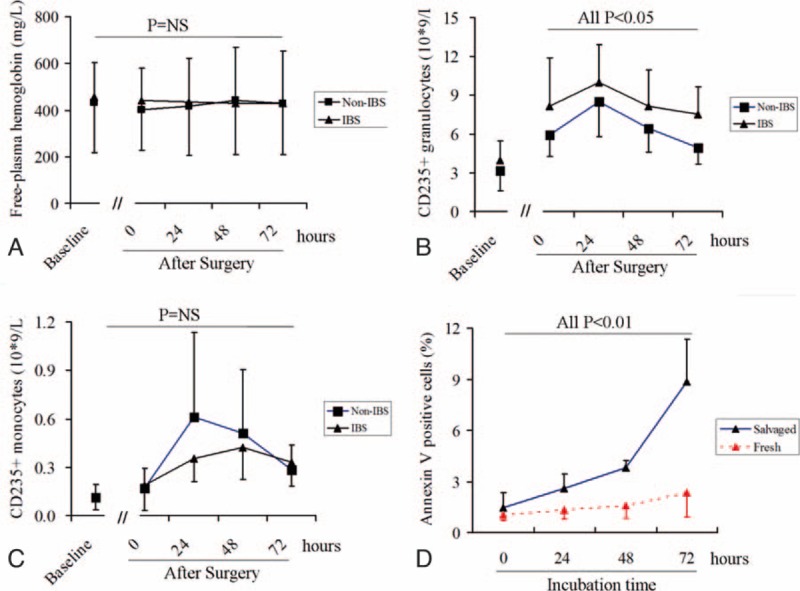
Comparison of (A) plasma-free hemoglobin levels, (B) CD235a-positive granulocytes, and (C) CD235a-positive monocytes in the 2 groups. (D) Increases in percentages of Annexin-V-binding cells over time from RBCs within the same patient. Salvaged and fresh erythrocytes from each of 10 patients were cultured (5 × 10^6^/mL) in fresh plasma for 72 hours in a 37 °C water bath on a shaker. NS = no significant difference.

Free plasma hemoglobin levels were similar between the 2 patient groups, and they remained stable throughout the observation period (Fig. 4A). Leukocyte counts in all patients increased after surgery, and counts were higher in the IBS group than in the non-IBS group. Flow cytometry showed similar results for counts of CD235a-positive granulocytes (Fig. 4B). In contrast, counts of CD235a-positive monocytes were similar between groups at all time points tested (Fig. 4C).

To examine whether IBS induces phosphatidylserine exposure, salvaged and fresh RBCs were isolated in the IBS group and compared within the same patient. The percentage of salvaged cells that bound Annexin-V was higher than the corresponding percentage of fresh cells from the same patient (2.04 ± 1.34% vs 1.45 ± 0.81%; *P* < .01). Incubating salvaged and fresh RBCs for 72 hours led to an increase in the percentage of Annexin-V-binding cells in a time-dependent manner. This increase was greater for salvaged cells than fresh cells by 1.48-fold at 0 hour of incubation, 3.62-fold at 24 hours, 2.73-fold at 48 hours, and 4.62-fold at 72 hours (Fig. 4D, all *P* < .001).

## 4 Discussion

Although IBS has been used for decades, several randomized controlled trials^[5–7]^ suggest that it does not reduce the requirement for postoperative RBC transfusion. This has led some to argue that IBS is not justified because the associated healthcare costs outweigh the demonstrated benefits.^[12]^ We did find, in the present study, that IBS helped maintain hemoglobin levels through the end of surgery, but it was associated with significantly reduced hemoglobin levels within 3 days after surgery. This may have been the cause of more than half of RBC transfusions within 3 days after surgery. These results suggest that IBS shortens the lifespan of salvaged RBCs.

Previous efforts to determine RBC lifespan have relied on RBCs labeled with biotin^[13]^ or the radionuclide ^51^Cr,^[14,15]^ neither of which is authorized for in vivo use in most countries. In addition, our approach allowed the detection of labeled RBCs even after engulfment by phagocytes (Fig. 1). It is unknown whether biotin and ^51^Cr are released or remain attached to RBCs after engulfment. This uncertainty may make previous studies of RBC lifespan less reliable than the results obtained with our method. Other medical centers should be able to verify and extend our findings in vivo.

Our study found that over 60% of blood lost was salvaged during surgery, which may explain why hemoglobin levels at the end of surgery were similar between groups, even though the volume of blood lost was 3-fold higher in the IBS group than in the non-IBS group. This suggests that IBS can effectively maintain hemoglobin levels perioperatively. However, while hemoglobin levels during the 3 days after surgery increased in the non-IBS group, they decreased in the IBS group. Furthermore, the volume of salvaged RBCs correlated with the magnitude of the hemoglobin decrease, and also with the nadir hemoglobin level. It was also identified as an independent risk factor for hemoglobin decrease. These results suggest that salvaged RBCs may be cleared rapidly after surgery.

In an effort to identify the mechanism(s) behind the lifespan-shortening effect of IBS on RBCs, we examined the possibility that IBS may induce hemolysis. We found that IBS was not associated with an increase in free hemoglobin levels in plasma, consistent with other studies.^[15,16]^ This suggests that hemolysis is not the main reason for shortened RBC lifespan.

As another possibility, we explored whether injured RBCs are engulfed by phagocytes via recognition of phosphatidylserine on the RBC membrane.^[10,11]^ We labeled RBCs based on the presence of CD235a, and phosphatidylserine was labeled with Annexin-V. The number of leukocytes increased after salvaged RBCs were returned to the body, and the number of RBC-engulfing granulocytes after surgery was significantly higher in the IBS group than in the non-IBS group. These findings suggest that IBS makes RBCs more likely to be engulfed after re-infusion, leading them to be cleared from the body.^[10,11]^ Indeed, we found that a higher proportion of salvaged cells than fresh cells had exposed phosphatidylserine on their surface, which acts as a signal for phagocyte engulfment. The proportion of salvaged cells with exposed phosphatidylserine was as high as 11% at 72 hours after blood salvage. In addition, numbers of CD235a-positive cells increased significantly within 72 hours after surgery in the IBS group. Therefore, we propose that at least 1 of the reasons why IBS does not reduce the postoperative requirement for blood transfusions is that it shortens RBC lifespan by increasing phosphatidylserine exposure on the RBC surface, facilitating engulfment of RBCs after re-infusion.

In our cohort, the proportion of patients undergoing surgery at sites of lumbar vertebra was higher in the IBS group than in non-IBS group, and surgery lasted slightly longer in the IBS group, with longer duration associated with massive blood loss. We found that hemoglobin decrease was associated with salvaged RBC volume, preoperative platelet count, and surgery site. Therefore, these parameters should be treated as potential confounders in studies of the impact of salvaged blood on hemoglobin levels.

Our results should be interpreted with caution in light of several limitations. The patients in our study were not randomized, but grouped according to whether they received IBS or not. As a result, differences among the 3 groups may have introduced bias into our results. Our study was limited by small sample size, which precluded us from analyzing whether IBS is an independent risk factor for postoperative transfusion of packed RBCs. We also did not examine whether different cell saver devices affect the function of salvaged RBCs, as previously reported.^[17]^ Finally, we did not measure ferritin, iron, erythropoietin, or reticulocyte counts, which would have indicated whether the patient had iron deficiency and would respond appropriately to blood loss.

## 5 Conclusions

Despite these limitations, the present study provides evidence that IBS shortens RBC lifespan, at least partly by increasing phosphatidylserine exposure on the RBC surface and facilitating engulfment of RBCs after re-infusion. Future studies should examine how to prolong the lifespan of salvaged RBCs, such as through changes to the RBC washing procedure to reduce injury during salvage, and modification of the washing solution to increase adenine nucleotide levels within the RBCs.^[9]^
